# Australian Inebriates Bill

**Published:** 1899-09-09

**Authors:** 


### AUSTRALIAN INEBRIATES BILL.

A Bill has lately come before Parliament in New South Wales, providing for the care, control, and treatment of inebriates much in the same manner as lunatics are dealt with. According to the proposed Bill it will be lawful for a judge of the Supreme Court, or of a District Court, or the master in lunacy, or any stipendiary or police magistrate, to order an inebriate to be placed under the care and control of some person named in the order, or of a hospital or licensed institution, for a period not exceeding 28 days; or in a licensed institution for such a period not exceeding 12 months as shall be mentioned in the order; or for the same period in the care and charge of an attendant or attendants, to be named in the order, and who shall be under the control of the judge, master in lunacy, or magistrate making the order. The order by which this process of seclusion can be put in force can be obtained on the application of the inebriate himself, or of any person authorised in writing in that behalf by the inebriate while sober; or of the husband, wife, or parent, or a brother, sister, son or daughter of full age, or a partner in the business of an inebriate; or of a member of the police force above the rank of sub-inspector, acting on the request of a duly qualified practitioner in professional attendance on the inebriate, or on the request of a relative of the inebriate, or at the instance of a justice of the peace; but proof must be given which shall satisfy the judge, magistrate, &c., that the person in respect of whom the application is made is an inebriate.

The granting of the necessary certificates and the reception of cases under the Act are guarded by restriction very similar to those in force in regard to lunacy cases. Besides the methods of committal to an institution already mentioned, where an inebriate has thrice within the preceding twelve months been convicted of an offence " of which drunkenness is a necessary ingredient," it is to be lawful for any Court of Petty Sessions to order that the inebriate be placed for not less than six nor more than twelve months "in any institution which may be established by the Government for the reception, control, and treatment of inebriates so convicted," and on the order of the judge or the master in lunacy " such period may from time to time be extended for further periods not exceeding twelve months each." No statement is made as to how many times such extensions may be repeated. There is a further provision that where the inebriate is physically unfit to travel to the institution named in the order, the Court making the order may direct that he be placed for immediate medical treatment in a gaol or lock-up, or hospital, or private house, under the supervision of the police.

There can be little doubt that an Act framed on these lines ought to be a very powerful engine for the suppression of habitual drunkenness. It is open to question, however, whether the English people would readily sanction any such interference with the liberty of inebriates who have not offended society in any other manner than by their over-indulgence. It is to be noted that in the Bill to which we now refer no attempt is made to define what is meant by inebriety, and we foresee great difficulties in framing any definition which shall practically meet the case, in view of the extremely varying character of the manifestations of drunkenness among different people.

In view of the great jealousy with which the public have always regarded any attempt to enlarge the power of control over lunatics we can hardly expect that still greater powers would be readily granted in the case of that ill-defined being " the inebriate." The first step in the control of inebriates obviously should be to control those in whom inebriety is shown to lead to or be associated with crime, and this is what we are now attempting in England; although it cannot be said that as yet much progress has been made since we got our Act. What is clear is that unless such an Act as that proposed in New South Wales were administered with considerable discretion, it might easily become an Act for the relief of families blessed with ne'er-do-well relations. Many a family would gladly place its reprobate member under control, if only for respectability's sake; especially if, as would seem to be the case, the impecunious inebriate would be the charge of the State. To let the reprobate drift into the workhouse is not respectable, and on the strength of this he sticks like a leech to his family. But an " institution " for his " reformation " would be quite another affair—and when he came out a few bottles of whisky would soon send him in again.

